# Multicentre Covariate Adjustment Analysis of Short-Term and 5-Year Outcomes after Endovascular Repair according to Sex

**DOI:** 10.1155/2020/8970759

**Published:** 2020-03-09

**Authors:** Bibombe P. Mwipatayi, Rebekah L. W Tan, Joseph Faraj, Ali Daneshmand, Olufemi Oshin, Nishath Altaf, Shannon D. Thomas, Patrik J Tosenovsky, Jackie Wong, Vikram Vijayan, Anthony J. Freeman, Sally A. Burrows

**Affiliations:** ^1^Department of Vascular Surgery, Royal Perth Hospital, Perth, WA, Australia; ^2^Faculty of Medicine, Dentistry and Health Sciences, University of Western Australia, Perth, WA, Australia; ^3^Perth Institute of Vascular Research, Perth, WA, Australia; ^4^Department of Vascular Surgery, Prince of Wales Hospital, Sydney, NSW, Australia; ^5^Faculty of Medicine, University of New South Wales, Sydney, NSW, Australia; ^6^The Vascular Institute, Prince of Wales Hospital, Sydney, NSW, Australia; ^7^Vascular Diagnostic Laboratory, Ng Teng Fong General Hospital, Singapore; ^8^Faculty of Medicine and Health, University of Sydney, Sydney, NSW, Australia; ^9^School of Medicine, University of Western Australia, Perth, WA, Australia

## Abstract

**Background:**

Several studies have reported worse outcomes in women compared to men after endovascular aneurysm repair (EVAR). This study aimed to evaluate sex-specific short-term and 5-year outcomes after EVAR.

**Methods:**

A total of 409 consecutive patients underwent elective EVAR from 2004 to 2017 at two tertiary hospitals in Western Australia. Baseline, intraoperative, and postoperative variables were examined retrospectively according to sex. The primary outcome was 30-day mortality (death within 30 days after EVAR). Secondary outcomes were 30-day composite endpoint, length of stay after EVAR, 5-year survival, freedom from reintervention, residual aneurysm size after EVAR, and major adverse event rate at 5-year follow-up.

**Results:**

A cohort of 409 patients, comprising 57 women (14%) and 352 men (86%), was analysed. Female patients were older (median age, 76.8 versus 73.5 years, *p*=0.017). Male patients were more likely to be past smokers (40.9% versus 22.8%, *p*=0.017). Male patients were more likely to be past smokers (40.9% versus 22.8%, *p*=0.017). Male patients were more likely to be past smokers (40.9% versus 22.8%, *p*=0.017). Male patients were more likely to be past smokers (40.9% versus 22.8%, *p*=0.017). Male patients were more likely to be past smokers (40.9% versus 22.8%, *p*=0.017). Male patients were more likely to be past smokers (40.9% versus 22.8%, *p*=0.017). Male patients were more likely to be past smokers (40.9% versus 22.8%,

**Conclusion:**

This study found no significant differences in 30-day and 5-year outcomes between female and male patients treated with EVAR, implying that EVAR remains a safe treatment choice for female patients.

## 1. Introduction

There is a higher prevalence of abdominal aortic aneurysms (AAA) in men than in women, with a 4 : 1 male predominance [1]. Intervention is usually offered when the aneurysm diameter has reached 5.5 cm or if the aneurysm is symptomatic. This consensus on size was established through various randomised controlled trials, wherein women were mostly underrepresented [2, 3].

Compared to men, women are often diagnosed at a later age, have a faster rate of aneurysm growth, a fourfold higher risk of rupture, and a higher risk of rupture at a smaller AAA diameter, and are three times more likely to die following the rupture of an aneurysm [4, 5]. Differences in pathophysiology, presentation, and outcomes following repair of AAA have been reported between sexes, rendering it difficult to identify the optimal threshold for treatment [6–8].

Endovascular aneurysm repair (EVAR) is increasingly performed as an alternative to open repair. EVAR is a minimally invasive treatment option for those deemed unfit for open surgery [9]. It is also associated with shorter hospital stays and reduced short-term mortality [9]; however, this benefit may not be the case in female patients who demonstrate a three-fold increase in mortality following elective EVAR compared to their male counterparts [10, 11].

The difference in these outcomes is poorly understood; however, several explanations have been postulated. Women tend to present at a more advanced age and more often with undiagnosed and untreated comorbidities that may increase their perioperative risk [1, 4]. In addition, women have been shown to have more adverse infrarenal and iliac anatomy that is associated with higher procedural complication rates, such as endoleak, abandonment of procedure, conversion to open surgery, and need reintervention [4, 6, 10]. Female patients also represent a minority of EVAR recipients in most trials; hence, our knowledge on EVAR and improvements made to operative devices are inherently based more on the male population. As such, the current AAA management guidelines are predominantly based on outcomes in male patients, and whether these outcomes should guide management in female patients is controversial. In this study, we sought to investigate the relationship between sex and outcomes following EVAR.

## 2. Methods

This is a retrospective study based on a prospectively collected database. The study subjects comprise all patients treated electively with an endovascular aortic repair for an AAA, at two major Western Australian hospitals, between January 2004 and December 2017. Patient data were retrieved from the hospital's electronic database and medical records where patients were followed in hospital clinics. Further, preoperative and control CTAs, angiographic findings, and ultrasound data were reanalysed. Patient inclusion criteria were age >18 years and indication for elective repair of AAA with an endovascular stent graft in accordance with the guidelines on endovascular aneurysm repair. Patients who had undergone thoracic endovascular aortic repair were excluded.

Baseline data including comorbidities were collected and stored in an electronic worksheet (Microsoft Excel, Palo Alto, CA). The primary outcome was 30-day mortality, defined as death within 30 days of the index EVAR. Secondary outcomes were 30-day composite endpoint (local and systemic complications, conversion to open repair, and occurrence of endoleak), length of stay (LOS) after EVAR between both groups, 5-year survival between both groups, freedom from reintervention at 5-years follow-up, residual aneurysm size after EVAR at 5-years follow-up, and major adverse event (MAE) rate at 5-years follow-up. Technical factors were also analysed, such as successful delivery and deployment of the stent graft in the absence of stent graft malfunction, kinking/twisting, or fracture. Information at follow-up visits (1, 3, 6, 12, and yearly thereafter) was recorded on a standardised electronic case record form.

MAEs were defined as either death or interventions including conversion to open repair, aneurysm rupture, major embolic event, graft infection requiring explanation, device migration, loss of device patency requiring reintervention, wound infection, and other complications (including cardiovascular, pulmonary, renal, gastrointestinal, and neurological complications). Device migration was reported if it required intervention or if an adequate seal was lost, usually when reduced to <10 mm of the circumferential apposition length. This study received ethical approval from the ethics committee of each institution. Informed consent was not sought as anonymised patient data were used.

### 2.1. Statistical Analysis

Counts and percentages, means and standard deviations or medians, and first to third quartiles [Q1, Q3] were used to summarise data. Differences in patient demographics and anatomical characteristics between male and female patients were tested using chi-square or Fisher's exact test for categorical characteristics; *t*-tests or Wilcoxon rank sum tests were used for continuous characteristics.

**Figure 1:**
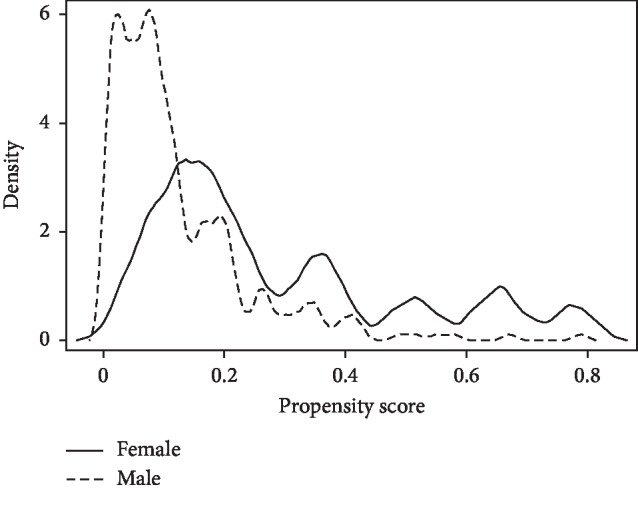
Propensity score model testing the assumption of overlap that each individual has a positive probability of receiving each treatment level.

Differences in mortality and MAEs between male and female patients were investigated initially using univariate logistic regression. Multivariable logistic regressions were then performed on 5-year outcomes to adjust for age, American Society of Anesthesiologists classification, comorbidities, hospital, stent type, and AAA characteristics, and bootstrapped to ensure that *p* values were robust to overfitting. A variance adjustment was applied to the regression models to account for clustering of patients within treating surgeons. Further, inverse probability-weighted analysis with a regression adjustment (propensity score) was performed to avoid overfitting. A propensity score model was tested in the assumption of overlap that everyone in the study had a positive probability of receiving each treatment level equally, but this suggested that the overlap assumption was not violated. This was more robust because it weighted the analysis using the propensity score in addition to the regression adjustment (Figure 1).

A Kaplan-Meier curve was generated to illustrate the unadjusted long-term survival function for male and female patients and differences tested using the log-rank test. Differences in hospital LOS were analysed using truncated negative binomial regressions due to the count nature of the outcome and skewness present. All analyses were performed using Stata 15 (StataCorp. 2017. *Stata Statistical Software: Release 15*. College Station, TX: StataCorp LLC), and significance was set at *p* < 0.05.

## 3. Results

From 2004 to 2017, 409 patients (57 women and 352 men) underwent elective EVAR. Baseline demographics and clinical characteristics are listed and compared in Table 1. During intervention, women were significantly older than men (mean age, 76.8 years versus 73.5 years, *p*=0.017). Most patients, regardless of sex, were Caucasian. Male patients were significantly more likely to be past smokers (40.9% versus 22.8%, *p*=0.005), have a history of coronary artery bypass grafting (CABG) (11.2% versus 3.5%, *p*=0.042), and have a history of malignancy compared with their female counterparts (24.1% versus 10.5%, *p*=0.014). No other significant differences in comorbidities or baseline characteristics were detected between sexes.

### 3.1. Anatomical Characteristics

The stent graft type is listed in Table 1. No statistical difference in device selection was found between the sexes. Anatomical details are summarised in Table 2. Whilst female patients had smaller infrarenal aortic neck angles (14.0° versus 20.2°, *p*=0.041) compared to that in men, no significant differences in aneurysmal sac diameter, length of infrarenal aortic neck, and the length of the aneurysm were noted between sexes. Neck calcification, thrombus load, and distal common iliac artery diameter did not reach a statistically significant difference between sexes.

### 3.2. Perioperative Outcomes

No significant differences in the average length of postoperative hospital or intensive care stays were observed between sexes (Table 3). No differences were found in technical success (98.2% versus 98.6%, *p*=0.597), freedom from intraoperative death (100% both groups), and freedom from type I endoleak (0.00% versus 0.85%). However, type II and III endoleaks were higher in male patients than in female patients (10.8% versus 7.0% and 0.0% versus 0.6%, respectively); nonetheless, these differences were not statistically significant between sexes. The estimated all-cause mortality rates at 30 days were 3.5% (*n* = 2) in women and 0.3% (*n* = 1) in men (*p*=0.052).

Complications occurring following surgery are shown in Table 3. Complication rates were slightly higher in female than in male patients, that is, cardiovascular events at 10.5% versus 7.7%, groin wound haematoma at 8.8% versus 4.8%, and wound infection at 5.3% versus 2.3%; conversely, access vessel complications, postoperative gastrointestinal bleeding, ischaemic colitis, and neurological accidents were more observed in men than in women. However, none of these differences were statistically significant between sexes.

### 3.3. Late Outcomes

**Figure 2:**
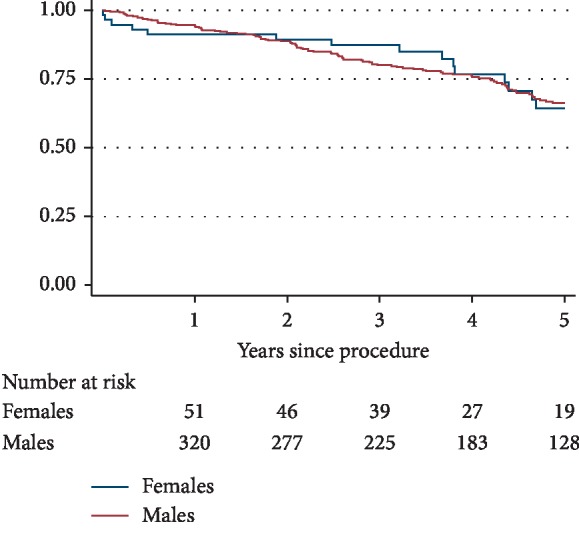
Five-year Kaplan–Meier survival curve by gender.

MAEs occurred in 20 (35.7%) women and in 123 (35.5%) men (*p*=0.981). At 5 years, 35 (87.5%) women and 222 (88.8%) men were free from arterial reinterventions (*p*=0.812). No significant difference was found in unadjusted long-term mortality between male and female patients (15 (26.8%) versus 95 (27.5%), *p*=0.907; odds ratio (OR) = 1.04; 95% confidence interval (CI), 0.53–2.02) which was also reflected in the Kaplan–Meier survival curve (log-rank test *p*=0.928) (Figure 2). Residual aneurysmal size assessed by ultrasonography did not differ significantly between the two groups (51.2 mm versus 53.2 mm, *p*=1.000).

The multivariate logistic regression demonstrated no difference in 5-year mortality for women compared with men (OR 1.07; 95% CI, 0.44–2.94; *p*=0.81) nor for the composite of mortality and MAEs at 5 years (OR 1.04; 95% CI, 0.93–1.18; *p*=0.6) (Table 4).

## 4. Discussion

This study found no difference in the short-term and long-term mortality and morbidity between male and female patients who underwent EVAR. This result supports findings from several series which acknowledge that, despite some differences in baseline characteristics between men and women, similar perioperative mortality and survival benefit exist; thus, EVAR should be recommended to suitable candidates regardless of their sex [12]. Contrary to this, other studies show that female patients have not experienced the same degree of positive outcomes after EVAR that male patients have had due to anatomical and biologic differences, delayed presentation, and under-representation in clinical trials [13, 14]. Grootenboer et al.'s [15] article based on the EUROSTAR registry demonstrated no difference in 30-day mortality between male and female patients, but significantly higher cumulative incidence of the composite endpoint mortality, systemic complications, or conversion (OR 1.32, 95% CI 1.05–1.66). In their survival analysis, even after adjusting for covariates, they also demonstrated a statistically significant higher rate of composite endpoint mortality and reintervention (hazard risk ratio 1.28, 95% CI 1.07–1.54). Women represented 7.2% of their cohort.

Our study contributes to the current literature as it demonstrates outcomes for a relatively large cohort of patients in two major tertiary centres in Western Australia. As such, the results are representative of the contemporary population treated in this region. The cohort studied is also comparable to those studied in previous literature: (i) the proportion of female patients in our study (16.2%) parallels that quoted in the literature (range 7.8%–21.6%) [16, 17] and (ii) the finding that female patients who undergo elective EVAR are older than male patients is also a consistent finding with previous literature (age range 73–78 years) [13, 16, 17].

Several factors contributed to why female patients present at an older age for aortic aneurysm repair. First, women are less likely to have imaging that may detect AAA either intentionally or incidentally. Most AAA screening programmes do not include women; for example, the United Kingdom aortic aneurysm screening programme only includes men age >65 years. No formal AAA screening guidelines or programs exist currently in Australia [14, 18]. The lack of incidental imaging of the aorta could also relate to either women having fewer comorbidities or undergoing fewer investigation for their comorbidities. Another reason to AAA being diagnosed later in women may be attributed to the loss of oestrogen, which is postulated to be protective against aneurysm development for women in their earlier reproductive years. This protection may be eliminated after menopause [15, 19]. This means that the disease process starts later in life among women, hence delaying their presentation; thus, repair is performed at a more advanced age. Despite female patients undergoing EVAR at an older age than male patients, our study has shown that this has not negatively affected the short- and long-term outcomes after EVAR.

Previous studies have identified that female patients have a more challenging anatomy [20] contributing to longer operative time, increased need for transfusion, and higher rates of complications including endoleak, false aneurysm formation, and wound infection [21]. This, however, is not evident in our study population. Factors such as neck angulation, size of the aneurysm neck, iliac diameter, tortuosity of iliac arteries, and aneurysmal sac diameter at the time of operation were comparable, with no significant differences between sexes identified except for female patients having less intrarenal aortic neck angulation. The congruity in EVAR outcomes in female and male patients could potentially be explained by the homogeneity in baseline anatomical characteristics. Our study thus emphasises the importance of careful selection of patients to undergo EVAR not only with regard to anatomical suitability but also to optimising treatment of pre-existing comorbidities to achieve the best outcomes following surgery.

Another factor identified by previous studies, as being of concern, is the rate of undiagnosed comorbid conditions in female patients that then increases operative mortality and morbidity [22]. In our cohort, male patients were more likely than female patients to be past smokers and have a history of CABG; however, both groups had otherwise similar baseline characteristics. In this study, both groups also had no significant differences in technical success, LOS, and postoperative complications in the short- and longer-term period. This perhaps suggests that EVAR, when carried out in a truly elective setting, allows for thorough assessment of pre-existing comorbidities and full medical optimisation before the procedure. In addition, the elective nature of surgery allows careful early planning and engagement of other allied health professionals in the recovery period.

Nevertheless, our study has several limitations. As a retrospective, nonrandomised review of a largely prospectively maintained database, there may be a potential selection bias. Although we adjusted our comparison for differences between the two groups, there are still potential confounding factors that were not adjusted, including information regarding medication and surgeon experience. As with previous studies, the female patient in this study are significantly older than the males (76.8 ± 9.5 years in female patient versus 73.5 ± 9.8 years in male patient, *p* value = 0.017) and the number of female patients in this study (*n* = 57) may have compromised detection of a significant difference, therefore given rise to the risk for type II error. Nonetheless, our dataset reveals extensive aneurysm-specific anatomy details not previously explored. Since our patients were recruited into this cohort, there has been considerable progress in the stent graft device industry in improving aortic endografts. Hence, modelling studies are needed to assess the effect of newer devices on outcomes and complications between sexes after EVAR.

In conclusion, in our contemporary patient population where the ratio of female to male patients is comparable to previous studies, we have failed to demonstrate differences in early and 5-year outcome after EVAR, mortality, freedom from secondary endovascular procedures, and freedom from MAEs between sexes. As with all EVAR series, long-term follow-up with more patients will determine whether these outcomes remain durable. Thus, EVAR still remains the treatment of choice in both sexes.

## Figures and Tables

**Table 1:** Patient demographics.

Characteristic	Females (*n* = 57)	Males (*n* = 352)	*p* value
Age, years	76.8 ± 9.5	73.5 ± 9.8	**0.017**
BMI > 30	9 (15.8%)	94 (26.7%)	0.066
Ethnicity			0.243
Caucasian	52 (92.8%)	347 (98.6%)	
Asian	—	4 (1.1%)	
African	—	—	
Maori	—	—	
ATSI	4 (7.1%)	—	
Others	—	1 (0.3%)	
Comorbidities			
Smoking			
Current	24 (42.11%)	146 (41.5%)	0.929
Former	13 (22.8%)	143 (40.9%)	**0.005**
Hypertension	41 (71.9%)	269 (76.6%)	0.447
Hyperlipidaemia	28 (50%)	177 (52.4%)	0.743
Diabetes	12 (21.0%)	84 (23.9%)	0.639
Cardiac	34 (59.6%)	177 (50.3%)	0.188
Angina	10 (17.5%)	60 (17.0%)	0.926
Arrhythmia	8 (14.0%)	62 (17.6)	0.496
CABG	2 (3.5%)	40 (11.2%)	**0.042**
CAS	3 (5.3%)	24 (6.8%)	0.652
CHF	2 (3.5%)	27 (7.7%)	0.215
MI	7 (12.5%)	53 (15.2%)	0.592
Renal	5 (8.8%)	57 (16.2%)	0.124
Carotid			
CVA	7 (12.3%)	35 (10.0%)	0.598
TIA	5 (8.8%)	28 (7.9%)	0.835
PVD	13 (22.8%)	94 (26.7%)	0.529
COPD	14 (24.6%)	98 (27.8%)	0.603
History of malignancy	6 (10.5%)	85 (24.1%)	**0.014**
ASA classification			0.445
Class I	1	3	
Class II	12	75	
Class III	38	209	
Class IV	6	64	
Stent type			0.764
1 Endurant	22 (38.6%)	159 (45.4%)	
2 Cook Zenith	3 (5.3%)	28 (8%)	
3 Gore Excluder	9 (15.8%)	56 (16%)	
4 Nellix®/AFX	3 (5.3%)	17 (4.9%)	
5 Talent	7 (12.3%)	33 (9.4%)	
6 Complex EVAR	13 (22.8%)	57 (16.3%)	
Hospital			
Public			0.116

*Note*. BMI: body mass index; ATSI: Aboriginal or Torres Strait Islander; CABG: coronary artery bypass grafting; CAS: coronary artery stenting; CHF: chronic heart failure; MI: myocardial infarction; CVA: cerebrovascular accident; TIA: transient ischaemic attack; PVD: peripheral vascular disease; COPD: chronic obstructive pulmonary disease; ASA: American Society of Anesthesiologists classification; Nellix®: endovascular aneurysm sealing system.

**Table 2:** Anatomical characteristics.

Characteristics	Females (*n* = 57)	Males (*n* = 352)	*p* value
Size of aneurysm (mm)	56.3 ± 9.9	59.7 ± 13.3	0.082
Length of infrarenal neck (mm)	29.3 ± 8.8	32.3 ± 13.1	0.191
Proximal neck diameter (mm)	24.0 ± 3.2	24.1 ± 2.8	0.799
Distal neck diameter (mm)	27.3 ± 2.7	26.7 ± 2.9	0.112
Neck angulation (°)	14.0 ± 15.7	20.2 ± 20.4	0.041
Neck angulation > 60°	2 (3.5%)	35 (9.9%)	0.081
Neck flaring > 1 mm	27 (47.4%)	140 (39.8%)	0.282
Neck thrombosis			0.566
None	36 (63.2%)	215 (61.0%)	
Mild	14 (24.6%)	109 (31.0%)	
Moderate	6 (10.5%)	26 (7.4%)	
Severe	1 (1.7%)	2 (0.6%)	
Length of infrarenal aorta (mm)	117.9 ± 10.8	121.6 ± 13.9	0.289
Right CIA length (mm)	59.8 ± 11.3	61.9 ± 12.8	0.237
Distal right CIA diameter (mm)	14.5 ± 6.6	15.3 ± 7.5	0.259
Right EIA diameter (mm)	9.3 ± 1.3	9.6 ± 1.4	0.113
Right iliac tortuosity			0.423
None	35 (61.4%)	183 (52.0%)	
Mild	7 (12.3%)	61 (17.3%)	
Moderate	12 (21.0%)	74 (21.0%)	
Severe	3 (5.3%)	34 (9.7%)	
Left CIA length (mm)	56.7 ± 10.7	57.1 ± 8.8	0.139
Distal left CIA diameter (mm)	13.6 ± 2.6	13.8 ± 2.9	0.456
Left EIA diameter (mm)	9.6 ± 1.5	9.8 ± 1.4	0.285
Left iliac tortuosity			0.650
None	29 (50.9%)	156 (44.3%)	
Mild	14 (24.6%)	104 (29.5%)	
Moderate	12 (21.0%)	70 (19.9%)	
Severe	2 (3.5%)	22 (6.2%)	
Neck calcification			0.459
None	43 (75.4%)	278 (79.0%)	
Mild to moderate	12 (21.05%)	70 (19.9%)	
Severe	2 (3.5%)	4 (1.1%)	
Patent lumbar vessels	51 (89.5%)	292 (82.9%)	0.193
Patent IMA	49 (86.0%)	286 (81.2%)	0.378
Patent left iliac	51 (89.5%)	333 (94.6%)	0.164
Patent right iliac	53 (93.0%)	326 (92.6%)	0.921

*Note*. CIA: common iliac artery; EIA: external iliac artery; IMA: inferior mesenteric artery.

**Table 3:** Perioperative data, 30-day, and long-term outcomes.

	Females (*n* = 57)	Males (*n* = 352)	*p* value
Type of anaesthesia			0.725
Local/regional	16 (28.1%)	91 (25.9%)	
General	41 (71.9%)	261 (74.1%)	
Length of stay (days)	6 (4,9)	4 (3,7)	0.279
Length of ICU stay (days)	1 (1,2)	1 (1,2)	0.896
Intraoperative			
Successful deployment	56 (98.2%)	346 (98.6%)	0.597
Death	—	—	
Rupture	—	5 (1.4%)	1.000
Conversion to open	1 (1.8%)	4 (1.1%)	0.530
Endoleak			
Type I	—	3 (0.85%)	1.000
Type II	4 (7.0%)	38 (10.8%)	0.362
Type III	—	2 (0.6%)	1.000
Type IV	—	1 (0.3%)	1.000
From operation to discharge			
Wound infection	3 (5.3%)	8 (2.3%)	0.187
Seroma	1 (1.7%)	7 (2.0%)	1.000
Haematoma	5 (8.8%)	17 (4.8%)	0.253
Access vessel dissection	—	4 (1.1%)	1.000
False aneurysm	—	3 (0.8%)	1.000
Distal vessel embolization	—	2 (0.6%)	1.000
Ischaemic limb	1 (1.7%)	4 (1.1%)	0.530
Ischaemic colitis	—	1 (0.3%)	1.000
CVS	6 (10.5%)	27 (7.7%)	0.478
Pulmonary	1 (1.7%)	12 (3.4%)	1.000
Renal	3 (5.3%)	19 (5.4%)	0.967
GI bleed	—	2 (0.6%)	1.000
CNS	—	3 (0.8%)	1.000
30-day mortality			
All cause	2 (3.5%)	1 (0.3%)	0.052
30-day composite outcome			
Complications and mortality	18 (31.6%)	98 (27.8%)	0.562
Long-term outcomes at 5 years			
All-cause mortality	15 (26.8%)	95 (27.5%)	0.907
Freedom from reintervention	35 (87.5%)	222 (88.8%)	0.812
MAE rate	20 (35.7%)	123 (35.5%)	0.981
Residual aneurysm size (mm)^†^	51.2 ± 17.2	53.2 ± 16.4	1.000

*Note*. ICU: intensive care unit; CVS: cardiovascular system; CNS: central nervous system; MAE: major adverse events. ^†^5 women and 51 men had residual aneurysm.

**Table 4:** Multivariable associations between baseline characteristics and 5-year mortality.

	Univariate			Multivariable		
	OR	95% CI	*p* value	OR	95% CI	*p* value
Sex	1.03	0.53–2.02	0.911	1.07	0.44–2.94	0.81
Age				1.09	1.05–1.11	<0.001
ASA physical status ≥3				0.63	0.28–1.39	0.26
Comorbidities						
Smoking				1.15	0.62–2.37	0.67
Hypertension				1.27	0.63–2.54	0.50
Angina				0.85	0.27–2.20	0.75
Arrhythmia				1.44	0.50–3.15	0.39
Coronary artery disease				1.12	0.54–2.35	0.75
Coronary artery bypass graft				1.48	0.37–4.33	0.50
Myocardial infarction				0.99	0.31–2.34	0.98
Chronic heart failure				3.23	0.87–10.97	0.09
Transient ischaemic attack				1.35	0.32–4.54	0.65
Cerebrovascular accident				0.85	0.24–2.41	0.79
Carotid artery stenting				1.52	0.33–5.23	0.52
Diabetes mellitus				1.68	0.75–3.40	0.16
Hyperlipidaemia				0.81	0.47–1.60	0.49
Peripheral vascular disease				1.09	0.56–2.49	0.82
Chronic kidney disease				0.76	0.25–1.87	0.56
COPD				1.36	0.67–2.59	0.38
Obesity				0.34	0.16–1.1	0.38
AAA characteristics						
Maximum AAA diameter (mm)				1.01	0.98–1.03	0.49
Distal neck diameter (mm)				0.94	0.81–1.09	0.42
Proximal neck diameter (mm)				1.08	0.93–1.28	0.36
Length of infrarenal neck (mm)				1.01	0.98–1.03	0.49
Composite mortality and MAE at 5 years				1.04	0.93–1.18	0.60

## Data Availability

Patient level data used for statistical analysis and to support the findings of this study are available from the corresponding author upon request.
